# Acknowledgment to Reviewers of *Viruses* in 2020

**DOI:** 10.3390/v13010098

**Published:** 2021-01-12

**Authors:** Viruses Editorial Office

**Affiliations:** MDPI AG, St. Alban-Anlage 66, 4052 Basel, Switzerland

Peer review is the driving force of journal development, and reviewers are gatekeepers who ensure that *Viruses* maintains its standards for the high quality of its published papers. Thanks to the cooperation of our reviewers, in 2020, the median time to first decision was 16 days and the median time to publication was 35 days. The editors would like to express their sincere gratitude to the following reviewers for their precious time and dedication, regardless of whether the papers were finally published:
A. McGargill, MaureenLetoha, TamásAbdallah, AliLeung, AnthonyAbdel Hakeem, MohamedLeung, Daisy W.Abdel-Mohsen, Mohamed T.Leung, Kwong-SakAbdel-Whab, El-SayedLeutenegger, Christian M.Abdelwhab, El-Sayed MLevin, JudithAbedon, StephenLevrero, MassimoAbergel, ChantalLevy, Ronald M.Abolnik, CeliaLewitus, EricABOUL-ATA, Aboul-AtaLeymarie, OlivierAbreu, CândidaLhomme, SébastienAccardi, LuisaLi, ChunfengAccotto, Gian PaoloLi, DapengAcharya, PriyamvadaLi, HaiAckermann, MathiasLi, HongminAdams, IanLi, JinquanAdams, Sandra D.Li, JonathanAdegbola, RaphaelLi, LinAdes, TonyLi, Melody HingAdkar-Purushothama, Charith RajLi, RenfengAdriaenssens, EvelienLi, ShitaoAggarwal, MeghaLi, XiangminAguilar-Carreno, HectorLi, XiaojunAherfi, SarahLi, YanliAhmad, AsrarLi, ZhuoranAiken, ChristopherLiang, ChenAirenne, KariLiang, Po-HuangAkatsuki, SaitoLiang, YuyingAkhmetzhanov, Andrei R.Liao, JiawenAkimov, SergeiLiao, Laura E.Akiyama, HisashiLiao, Yu-ChiehAlabi, OlufemiLiberski, Paweł P.Alam, SaminaLichterfeld, MathiasAlazem, MazenLim, Chun ShenAlbarnaz, J. D.Lim, Siew PhengAlbrecht, RandyLim, Stephanie MAlejo, AlíLin, Chao-NanAlekseev, KonstantinLin, Cheng-WenAlfieri, CarolineLin, TaoAli Zaidi, ShanLindberg, A. MichaelAllard, BenoitLindemann, DirkAlley, Maurice R.Lindenbach, BrettAllison, AndrewLing, ChenAllison, Heather E.Ling, JiaxinAlmeida, GabrielLing, JiqiangAlmendral, JoséLingwood, DanielAlonso, CovadongaLinial, MaxineAlonso, MartaLinial, Maxine L.Alphonse, SebastienLipińska, AndreaAltamura, GennaroLittlejohn, MargaretAltan-Bonnet, NihalLiu, GuanQunAly, HusseinLiu, HaolinAlymova, Irina V.Liu, Hsin-FuAmarasiri, MohanLiu, JiaAmarilla, Alberto A.Liu, QiangAmbagala, ArunaLiu, Shan-LuAmiri, EsmaeilLiu, WeiAmoutzias, GrigorisLiu, XiaoyingAn, Dong SungLiu, YueAn, Dong-JunLiu, ZhuomingAnany, HanyLizano, MarcelaAnaya-López, Jose LuisLizarazo, Erley F.Anda, BaicusLlanes, AlejandroAnderson, DanielleLlorente, FranciscoAnderson, JohnLo Giudice, RobertoAndersson, BjornLo, Shih-YenAndo, SugihiroLo, SzechengAndrás, IbolyaLoc-Carrillo, CatherineAndreoni, Kenneth A.Lodmell, J. StephenAngelova, Assia L.Logue, JamesAnis, EmanLohmann, VolkerAnsaldi, MireilleLojkić, IvanaAnticoli, SimonaLondon, Robert E.Anton, HalinaLondono, BerlinAntonyuk, SvetlanaLondrigan, SarahAntson, FredLópez, Carolina B.Antunes, Francisco José NunesLopez-Ferber, MiguelAparicio, FredericLópez-Ferber, MiguelApolonia, LuisLorenzo, GemaAquaro, StefanoLorusso, AlessioArakelyan, AnushLos, MarcinAralaguppe, Shambhu G.Lotz, JeffreyAranda, Miguel A.Louis, Irina St.Aranday-Cortes, ElihuLovell, ScottAraniciu, CătălinLowe, DavidArbuthnot, PatrickLozach, Pierre-YvesArduino, Roberto C.Lozano, Jose ManuelArens, RamonLozano-Durán, RosaArgaw, TakeleLu, TzupinArhel, Nathalie J.Lucifora, JulieArias, ArmandoLühken, RenkeArias, CarolinaLukacher, AronArien, Kevin K.Lukashevich, IgorArii, JunLukhovitskaya, NinaArkin, IsaiahLulla, ValeriaAronson, JudyLundgren, MagnusArriagada, GloriaLundstrom, KennethAsante, EmmanuelLuo, ZhenAsgari, SassanLupberger, JoachimAshour, HossamLüscher, BernhardAston, Emily J.Lustig, YanivAtanasova, KalinaLuthra, PriyaAtkinson, Sarah C.Luttermann, ChristineAttias, JosephLy, HinhAtwood, WalterLycett, SamanthaAudet, JonathanLynch, RebeccaAudsley, MichelleLynch, Stacey EAuvinen, EevaLyu, YuanAvinoam, OriMa, WenjunAvrani, SaritMabashi-Asazuma, HideakiAwasthi, SitaMacchi, BeatriceAzab, WalidMacdonald, AndrewAzam, Aa HaerumanMacDuff, Donna A.Azar, Sasha R.Macfarlan, ToddAzzi, AlbertaMachado, AlexandreBaaijens, JasmijnMaciejewska, BarbaraBabiuk, ShawnMaciver, SutherlandBabkin, Igor V.MacNeill, AmyBacharach, EranMaeda, KazuhikoBachmann, Martin FMaeda, NaoyoshiBagasra, OmarMaekawa, ShunBagchi, ParikshitMager, Dixie L.Bagiu, Iulia-CristinaMaggi, FabrizioBaigent, SueMaggirwar, SanjayBailey, Adam LeeMaghsoodi, AmenehBailey, DalanMaginnis, MelissaBailly, BenjaminMagor, KatharineBailly, Jean LucMahar, JackieBalachandran, SiddharthMahony, Timothy JohnBalaraman, VelmuruganMaillard, Pierre VBalasch, MonicaMaisetta, GiuseppantonioBalestrieri, EmanuelaMaixner, MichaelBálint, ÁdámMajerciak, VladimirBalkema-Buschmann, AnneMajewski, JacekBalla, Keir M.Majkowska-Skrobek, GrażynaBallegeer, MarliesMak, Lung-YiBamford, ConnorMakarova, Kira SBanadyga, LoganMakowski, LeeBańbura, Marcin W.Malagon, FranciscoBandea, ClaudiuMaldarelli, FrankBanerjee, Nilam SanjibMaldonado, JuanBánfalvi, ZsófiaMamede, JoaoBao, XiaoyongMang, Stefania MirelaBara, JeffreyMangala Prasad, VidyaBarbeau, BenoitMangino, GiorgioBarbezange, CyrilManicassamy, BalajiBarbic, LjuboMans, JanetBarbirz, StefanieManuel Giorgi, FedericoBarjesteh, NedaManzoor, RashidBarklis, EricMarc, DanielBarr, Kelli L.Marcacci, MauriliaBarrett, DamienMarchant, DavidBarril, XavierMarchetti, MoniaBarrs, VanessaMarchini, AntonioBartee, EricMarchiò, SerenaBartholomeeusen, Dr. KoenMarenzoni, Maria LuisaBártolo, InêsMargina, DenisaBartowsky, EvelineMargulies, BarryBarzon, LuisaMarin Lopez, AlejandroBattesti, AureliaMariner, JeffreyBaur, AndreasMaringer, KevinBausch, DanielMarjomaki, VarpuBaxter, Victoria K.Mark S., JohnsonBay, R CurtisMarkovich, Michal PerryBaz, MarianaMarkussen, TurhanBażanów, BarbaraMarquet, RolandBeatty, Julia AnneMarriott, AnthonyBeauvais, Wendy A.Marschall, ManfredBeaver, John R.Marsden, MatthewBecher, PaulMarston, DeniseBecker, Stefanie ChristineMarszałek, JarosławBeckham, J. DavidMartella, VitoBeckham, John DavidMartikainen, MiikaBecking, ThomasMartín Castillo, MargaritaBeerens, NancyMartin, AnnetteBehar, AdiMartin, EileenBehrens, Sven-ErikMartin, JavierBehzadi, PayamMartin, StephenBejarano, Eduardo R.Martin, Tammy M.Bejerman, NicolasMartín-Acebes, Miguel A.Bell, BrendanMartinet, Jean-PhilippeBelliot, GaëlMartinez, Jose CBellizzi, AnnaMartínez, Miguel A.Belogurov, GeorgiMartínez-García, ManuelBelov, GeorgeMartinez-Gil, LuisBelser, JessicaMartínez-Martínez, IreneBelsham, GrahamMartins, NelsonBelykh, OlgaMarty, GaryBenaroch, PhilippeMarutescu, LuminitaBender, Scott C.Marutescu, Luminita GabrielaBenest, AndrewMaruyama, JunkiBenfield, David A.Marzano, Shin-YiBénichou, SergeMas, AntonioBenitez, LauraMasalova, Olga V.Benítez, María JoséMasataka, TsugeBennett, AntonetteMassanella, MartaBennett, Richard SMastino, AntonioBen-Shachar, RotemMasuda, TakaoBergström, TomasMatalka, Khalid Z.Beringue, VincentMatczuk, Anna KarolinaBerkhout, BenMateo, MathieuBernacchi, SerenaMathew, AnujaBernatchez, Jean A.Mathieu, CyrilleBerrens, RebeccaMathijs, ElisabethBertho, NicolasMatsuo, EikoBerthon, PatriciaMatsushita, MichikoBertin, SabrinaMattapallil, JosephBertoni, GiuseppeMatthews, LouiseBertzbach, Luca DaniloMatusali, GiuliaBest, Simon R.A.Mauffret, OlivierBeyer, WolfgangMavian, CarlaBhaskar, SonuMay, JaredBhattacharjee, Apurba KrishnaMazurov, Dmitriy VBi, XiuqiongMazzei, MaurizioBibby, DavidMbonye, UriBickerton, EricaMcBride, JohnBicout, DominiqueMcCauley, MicahBideshi, DennisMcCormick, CraigBiedenkopf, NadineMcCulloch, KarenBielefeldt-Ohmann, HelleMcDonnel, Samantha J.Bienzle, DorotheeMcFadden, GrantBierle, CraigMcFarlane, Donald A.Biernat, BeataMcIlroy, DorianBigarré, LaurentMcInerney, GeraldBiliavska, LiubovMcKee, CliftonBillerbeck, EvaMcknight, AineBinder, MarcoMcLean, GaryBingham, JohnMcLean, RebeccaBisaillon, MartinMcMillan, NigelBishop-Lilly, Kimberly A.McVey, ColinBissonnette, NathalieMedina Piles, VicenteBiswas, SantanuMedina, Gisselle N.Biswas-Fiss, Esther E.Medina-Kauwe, Lali K.Black, LindsayMediouni, SoniaBlackstone, NeilMee, Edward T.Blacquière, TjeerdMeertens, LaurentBlahak, SilviaMehedi, MasfiqueBlair, CarolMehta, Sanjay R.Blair, Geoger EricMei, Ya-FangBlaise-Boisseau, SandraMeier-Stephenson, VanessaBlanchard, YannickMelcher, UlrichBlanco-Melo, DanielMelikian, GregoryBlasdell, KimMeliopoulos, Victoria ABlazquez, Ana-BelenMelzer, Michael J.Blutt, Sarah ElizabethMena, IgnacioBock, C. ThomasMencia Caballero, MarioBodem, JochenMendoza, EmelissaBoehme, Amelia K.Menéndez-Arias, LuisBoete, ChristopheMeng, XiangchaoBogers, Willy M J MMenne, StephanBoglione, LucioMenzo, StefanoBoissot, NathalieMergia, AyalewBojko, JamieMerino, Jose JoaquinBollyky, PaulMerkling, Sarah HeleneBolshoy, AlexanderMesci, PinarBonnefoy, ElietteMeškys, RolandasBono, HidemasaMesri, EnriqueBoon, Adrianus C. M.Metifiot, MathieuBopegamage, ShubhadaMetz, StefanBorgmann, KathleenMetz, Stefan W.Born, Erwin Van DenMetzger, MichaelBorodovsky, MarkMetzner, KarinBoroń-Kaczmarska, AnnaMeurens, FrancoisBorrego, BelénMeurs, ElianeBorsetti, AlessandraMeuti, MeganBorucki, MonicaMeyer, FlorenciaBosco, AnthonyMeyer, GillesBosco-Lauth, Angela MMeyer, HermannBosco-Lauth, Angela M.Meyer, JustinBošková, VeronikaMeyers, CraigBosse, Jens B.Miao, QiuhongBostina, MihneaMichaux, J. R.Botella Sánchez, LeticiaMichon, ThierryBoudreau, JeanetteMiesen, PascalBoulanger, PascaleMilani, AdelaideBoulant, SteeveMilani, MarioBouřa, EvženMilavetz, BarryBourgoin, SandrineMillard, AndrewBourquain, DanielMillard, Andrew D.Bourret, VincentMiller, W. AllenBowen, RichardMiller-Saunders, KristiBoyer, ThomasMillet, Jean K.Brackney, Douglas E.Minematsu, ToshioBradley, ToddMirabelli, CarmenBrai, AnnalauraMiranda, CarlaBrandt, CurtisMiroslaw, SnieturaBrandt, SabineMishra, AwdheshBrasier, Allan R.Mitsuru, OkuwakiBrenner, Bluma G.Miyashita, ShuheiBressanelli, StéphaneMiyazawa, MasaakiBreyta, RachelMizukami, ShusakuBreyton, CécileMlera, LuwanikaBrien, JamesMocarski, EdwardBrindley, MelindaMochizuki, TomofumiBrinkmann, AnnikaModahl, CassandraBrinton, MargoModha, SejalBrisse, MorganMoens, Ugo LionelBrnić, DraganMohanty, SmitaBrocato, RebeccaMöhl, BrittaBrochot, EtienneMohr, Emma L.Broecker, FelixMohsen, MonaBrookes, SharonMok, Chris C.P.Brookes, Sharon MMolineux, IanBrown, Amanda M.Monguió-Tortajada, MartaBrown, Ashley N.Monne, IsabellaBrown, KatherineMontagutełłi, XavierBrown, Paul AMontassier, Helio J.Brown, Richard J.P.Montes, NuriaBrown, TeaganMontoya, MariaBrowne, Edward P.Moore, Nicholas M.Bruce, Emily A.Moore, PatrickBruin, Erwin DeMorabito, Kaitlyn M.Brum, Jennifer R.Moraes, TheoBrune, WolframMorais, MarcBrutscher, LauraMoraru, Liliana CristinaBryan S., KaplanMordecai, GideonBryant, BartMoreno, MiguelBryden, StevenMorgan, EthanBrzozowska, EwaMorgan, Ethan L.Buchanan, ToreMori, YasukoBuchko, Garry W.Morimoto, KinjiroBuck, Christopher B.Morita, EijiBuening, HildegardMorley, ValerieBujarski, Józef J.Morozov, IgorBull, JimMorozova, OlgaBurckhardt, ChristophMorozova, Vera V.Burdo, Tricia H.Morris, MhairiBurges, GrahamMorrison, JulietBurrone, Oscar RobertoMorrison, Thomas E. "Tem"Burwitz, Benjamin J.Moss, BernardBuseyne, FlorenceMossel, EricButtimer, ColinMostafa, AhmedByadgi, Omkar VijayMousa, Jarrod J.Byrne, AlexanderMoutailler, SaraCabezon, OscarMoutelíková, RomanaCadar, DanielMucker, Eric M.Caddy, SarahMühlberger, ElkeCafiero, MauricioMukhopadhyay, TuliCagliani, RacheleMulder, LubbertusCagno, ValeriaMulkey, SarahCai, YingyunMullarkey, CaitlinCalderaro, AdrianaMüller, BarbaraCalendar, RichardMuller, David AlexanderCalistri, AriannaMüller, JanisCalistri, PaoloMuller, MandyCaller, LauraMunk, CarstenCalvignac-Spencer, SébastienMuñoz-Barroso, IsabelCalzolari, MattiaMuntean, Alexandru AndreiCamarasa, María-JoséMurakami, HironobuCambillau, ChristianMurakami, KosukeCamero, MicheleMurakami, ShinCamp, Jeremy V.Murakami, YoshikiCampbell, Corey L.Muramatsu, MasamichiCampbell, EllsworthMurata, TakayukiCampbell, Ellsworth M.Muriaux, DelphineCampisciano, GiusiMurillo, RosaCampos, Fabrício S.Murota, KatsunoriCampos, Rafael KroonMurovska, ModraCamus-Bouclainville, ChristelleMurovska, Modra F.Candela, Monica G.Murphy, Alex MCannon, PaulaMurphy, EainCantore, AlessioMurray, Shannon M.Canuti, MartaMusalgaonkar, SharmishthaCao, DianjunMustafa, GhazalaCara, AndreaMuthu Karuppan, Mohan KumarCardenas-Garcia, StivalisMutoloki, StephenCardeti, GiusyMuxel, Sandra MarciaCardin, RhondaMyers, DeanCareem, FaizalMyung, HeejoonCarey, ClaytonNachbagauer, RaffaelCarlton, JeremyNaesens, LieveCarmona-Vicente, NoeliaNagafuchi, SeihoCarnaccini, SilviaNagai, MakotoCarnahan, Robert H.Nagasaka, KazunoriCaron, AlexandreNaghavi, Mojgan H.Carow, BeritNaguib, MahmoudCarpentier, DavidNaik, ShruthiCarr, Jillian M.Naji, Hassan S.Carter, Carol A.Nakagawa, KojiCaruso, CalogeroNakagawa, SoCaruso, StevenNakahara, Kenji S.Casartelli, NicolettaNakahara, TomomiCasasnovas, Jose MaríaNakakita, Shin-ichiCase, James BrettNakanishi, AkiraCasjens, SherwoodNakauchi, MinaCasola, AntonellaNakayama, EriCastanha, Priscila M Da SilvaNakayashiki, HitoshiCastelletti, NoemiNakazawa, YoshinoriCastillo, DanielNanbo, AsukaCastle, AlanNasar, FarooqCatalão, Maria JoãoNavarro, BeatrizCaughey, ByronNavas-Martin, SoniaCella, EleonoraNavratil, VincentCerny, JanNawrocki, Steffan T.Cetinel, SibelNawrot, RobertChabannes, MatthieuNayak, TapanChambers, Joseph ENazli, AishaChambers, ThomasNeerukonda, SabarinathChandra, NareshNejman-Faleńczyk, BożenaChandrashekar, AbishekNekhai, SergeiChang, ChungjanNelson, Scott W.Chang, Hui-WenNemes, KatalinChang, JaewonNerva, LucaChang, PengxiangNetherton, Christopher L.Chang, Poa-ChunNeufeldt, Christopher JohnChang, Yen-ChenNeuman, BenjaminChao, Day-YuNeumann, DonnaChao, MeiNevola, RiccardoChao, Yu-ChanNice, TimothyChapman, Nora M.Nicoletti, LoredanaChapuy-Regaud, SabineNiedźwiedzka-Rystwej, PaulinaCharleston, MichaelNielsen, Tue KjærgaardCharon, JustineNiepmann, MichaelCharvat, RobertNiespodziana, KatarzynaChase, ChristopherNigam, DeeptiChattopadhyay, SaurabhNiikura, MasahiroChatzopoulou, FaniNishigaki, KazuoChaurasiya, ShyambabuNishihara, HidenoriChauveau, LiseNishizono, AkiraChavez-Calvillo, GabrielaNisole, SébastienChawla-Sarkar, MamtaNissimov, JozefChazal, NathalieNistal-Villán, EstanislaoChebloune, YahiaNjeumi, FelixChemin, IsabelleNofemela, Robert S.Chen, BingNogales, AitorChen, ChunhongNogalski, MaciejChen, FengNoguchi, YoshihiroChen, Hui-ChenNogueira, MauricioChen, Li LiNoris, EmanuelaChen, Shi-JieNovacek, JiriChen, Wei-JuneNovelli, GiuseppeChen, Yanping (Judy)Novosel, DinkoChen, YewangNowotny, NorbertCheng, HailiNuesch, JurgCheng, XiaomingNüesch, JürgCheng, XiaowenNugen, SamCheng, Xiao-WenNunberg, JackCherepanov, PeterNunez, AlejandroCheshenko, NataliaNúñez, José IgnacioChesnais, QuentinO'Connor, ChristineChe-Yen Wang, JosephOde, HirotakaChiappinelli, KatherineOdendall, CharlotteChiapponi, ChiaraOesterle, PaulChiba, SotaroOfferdahl, Danielle K.Chibo, DorisOgasawara, NorikoChico Gras, VerónicaOgden, KristenChihade, JoeOgembo, JavierChikh Ali, MohamadOgino, TomoakiChinnakannan, SenthilOhshima, TakayukiChira, SergiuOka, TomoichiroChiu, ChristopherOkabayashi, TamakiChiumenti, MichelaOkamoto, HiroakiChlanda, PetrOkamoto, ShigefumiCho, Jae-HyunOkayama, AkihikoCho, Nam-HyukOkazaki, KatsunoriCho, Tae-JuOksanen, HannaCho, Won KyongOlagoke, OlusolaChoi, Tae-JinOlds, Cassandra L.Choi, Young-KiOlech, MonikaChomont, NicolasOlejniczak, MartaChow, Yen-HungOlejnik-Schmidt, AgnieszkaChowdhury, RatulOlety, BalajiChowdhury, ShafiqulOliver, JoAnneChowell, GerardoOlson, KennethChristiaens, OlivierOng, David S.Y.Christian, Kimberly MOnomoto, KojiChristie, GailOomens, Antonius (Tom)Chromy, DavidOpekun, Antone RobertChua, Kaw BingOrchard, RobertChulanov, VladimirOrlova, Elena V.Chulanov, VladmirOrłowska, AnnaChung, DonghoonOrnelles, DavidChung, Hui-MinOrrú, ChristinaCiccozzi, MassimoOrtega, Joseph ThomasCilia, GiovanniOrtego, JavierCillo, FabrizioOscar D., SalomónCimica, VelascoOsna, NataliaCimolai, NevioOsolodkin, DmitryCinek, OndrejOSSIBOFF, Robert J.Cingolani, GinoOttmann, MichèleCiotti, MarcoOtulak-Kozieł, KatarzynaClarke, PennyOuwendijk, Werner J. D.Claverie, Jean-MichelØynebråten, IngerClement, SophieOzawa, MakotoCliffe, AnnaOzbun, MichelleClifford, David B.Pacenti, MoniaClouthier, SharonPacheco, BeatrizCobbin, JoannaPacioni, CarloCobo, Jordi SerraPaczesny, JanCocklin, SimonPadhi, Bhaja KrushnaCoffin, John M.Paes, Marciano VianaCohen, DanielPaeshuyse, JanCohrs, RandallPagán, IsraelColavita, FrancescaPage-Karjian, AnnieColler, Beth-annPaillart, Jean ChristopheCollisson, EllenPaillot, RomainColpitts, Che C.Paixão, Enny S.Comas-Garcia, AndreuPajkrt, DasjaComas-Garcia, MauricioPalamara, Anna TeresaCombet, ChristophePalinski, RachelCompeer, Ewoud B.Palmenberg, Ann C.Compton, AlexPalukaitis, PeterConaldi, Pier GiulioPandey, Krishan K.Confer, Anthony W.Pandit, AridamanCong, YingyingPanfil, AmandaCongBao, KangPanganiban, AntonitoConnerton, IanPanicz, RemigiuszCoombs, KevinPantůček, RomanCooper, CallumPapin, James F.Corcoran, JenniferPappu, HanuCorcoran, Jennifer APaquin-Proulx, DominicCork, SusanParadkar, PrasadCorrea, MaríaParan, NirCortese, MirkoParcells, MarkCortez, ValerieParissi, VincentCorti, ManuelaPark, EujinCortjens, BartPark, JunsooCory, Theodore J.Parks, GriffithCosby, LouiseParolin, CristinaCosseddu, Gian MarioParra, GabrielCosset, François-LoïcParreira, RicardoCosta, Ana RitaParrish, ColinCostafreda Salvany, Maria IsabelParsons, MatthewCostantini, Veronica P.Pascal, Steven M.Costanzo, MichelePascalis, HervéCôté, MarcelinePasdeloup, DavidCotelle, PhilippePaskaleva, ElenaCotten, MatthewPassaes, Caroline Pereira BittencourtCouderc, ThérèsePassler, ThomasCoulibaly, FasseliPastorek, JaromirCox, Andrea L.Patel, Trushar R.Cox, RobertPathak, Vinay K.Craig, MorganPatil, BasavaprabhuCrawford, LindseyPatterson, StevenCreager, HannahPatterson-West, JenniferCrepin, ThibautPaulauskas, AlgimantasCrisci, ElisaPaupy, ChristopheCrocchiolo, RobertoPavelko, Kevin D.Croft, LarryPavesi, AngeloCrook, BrianPavlov, KonstantinCsorba, TiborPawlak, AleksandraCuesta, AlbertoPaxton, RobertCui, HongyuPayne, SusanCullen, BryanPazgier, MarzenaCun, WeiPearson, AngelaCusi, Maria GraziaPECHEUR, EveCynis, HolgerPeci, AdrianaCzosnek, HenrykPedersen, Finn SkouD’Angelo, AlbertoPedrera, MiriamDa Costa, Rui M. GilPeeters, Ben P. H.Da Fonseca, Flavio GuimaraesPegan, Scott D.Dabrowska, KrystynaPei, JingjingDacheux, LaurentPei, YonggangDahle, Maria KPeitsch, Manuel ClaudeDalal, VijitPelchat, MartinDalgaard, Tina SørensenPelka, PeterDalianis, TinaPena, Lindomar JoséDalmon, AnnePenades, JoseDaly, JanetPeng, ChenDamtie, DebasuPenna, ClaudiaD'Andrea, Marco MariaPénzes, JuditDarpel, KarinPerdoni, FedericaDas, KalyanPereira, CarlaDastjerdi, AkbarPereira, RobertoDatta, SiddharthaPerelson, AlanDaugrois, Jean-HeinrichPérez Artés, EncarnaciónDavido, DavidPerez Sepulveda, BlancaDavidson, AndrewPerez, DanielDavidson, IritPérez, Lester J.Davies, Bryan WilliamPerez-Sautu, UnaiDavis, April D.Persson, IngmarDavis, David A.Peterlin, BorisDavydova, JuliaPetraityte-Burneikiene, RasaDawson, Erica D.Petráš, MarekDe Alwis, Ruklanthi (Rukie)Petrillo, MarcoDe Benedictis, PaolaPetrov, AntonDe Bernardi Schneider, AdrianoPetruzziello, ArnolfoDe Breyne, SylvainPetry, KlausDe Chiara, GiovannaPetrzik, KarelDe Clercq, ErikPettersson, JohnDe Francesco, RaffaelePettersson, UlfDe Gascun, Cillian F.Peyambari, MahtabDe Graaf, MirandaPfaender, StephanieDe Groof, AdPfaller, ChristianDe La Pena, MarcosPfeffer, Martinde la Torre, Juan C.Phalen, DavidDe Marco, Maria AlessandraPhan, TungDe Miranda, Joachim R.Piancatelli, DanielaDe Noronha, CarlosPiantadosi, AnneDe Paola, DomenicoPiazza, RoccoDe Regge, NickPichat, PierreDe Souza, William MarcielPierangeli, AlessandraDe Swart, RikPietersen, GerhardDe Toni, LucaPietrancosta, NicolasDe Vos, DanielPijlman, GorbenDe Vries, Rory D.Pikula, AnnaDeback, ClairePilalas, DimitriosDebadyuti, GhoshPilotto, MarianaDeboutte, WardPincus, SethDebyser, ZegerPingarilho, MartaDeCaprio, JamesPintel, DavidDecaro, NicolaPinto, Amelia K.Decker, AldoPinto, Rosa MDeFilippis, Victor R.Pipas, James M.Deim, ZoltánPires, DianaDel Fresno Sánchez, CarlosPischke, SvenDel Real, GustavoPistello, MauroDel Valle, LuisPittau, MarcoDelhon, GustavoPizzorno, MarieDelhon, Gustavo A.Plattet, PhilippeDella Bartola, MichelePleckaityte, MildaDelputte, PeterPlemper, RichardDelwart, EricPleshakova, Tatyana O.Demangeat, GérardPöhlmann, StefanDembowski, JillPokorski, MieczyslawDeng, XufangPolak, Mirosław PawełDeniziak, StanisławPolonis, VictoriaDenzin, NicolaiPolyak, StephenDepaquit, JeromePolz-Dacewicz, MałgorzataDesbiez, CécilePomar, LeóDesprès, PhilippePompon, JulienDesselberger, UlrichPoncet, DidierDestainville, NicolasPonraj, PrabakaranDeStefano, JefferyPonsero, Alise J.Deutskens, FabianPonz, FernandoDevaux, PatriciaPop, Tudor LucianDeviatkin, Andrei A.Popham, Holly J.Dhakal, SantoshPossee, RobertDhungel, PragyeshPotapov, Sergey A.Di Bella, SantinaPoteat, ToniaDi Gennaro, FrancescoPoterszman, ArnaudDi Giallonardo, FrancescaPotokar, MajaDi Martino, BarbaraPoulet, HervéDi Micco, SimonePoulicard, NilsDi Nunzio, FrancescaPouliopoulos, AntonisDíaz Mochón, Juan JoséPoulson, RebeccaDiaz, ArturoPowers, AnnDiaz-Griffero, FelipePowis, Simon JohnDiaz-San Segundo, FaynaPrabakaran, MookkanDick, RobertPradines, BrunoDiederich, SandraPrevelige Jr., Peter E.Dieterle, María EugeniaPreziuso, SilviaDietrich, IsabellePrice, PatriciaDietrich, UrsulaPrischi, FilippoDietzgen, RalfPriyamvada, LalitaDijkman, RonaldProdělalová, JanaDikeakos, JimmyProkhorov, NikolaiDimienescu, Oana GabrielaProsperi, AliceDimitrov, KirilProverbio, DanielaDimitrov, Kiril M.Puchol, Sandra MartínezDineley, KellyPuertas, Maria CarmenDing, Shou-WeiPuff, ChristinaDiskin, RonPuig-Barberà, JuanDobbler, GerhardPullan, Steven T.Dodds, W. JeanPunga, TanelDokland, TerjePuray-Chavez, Maritza N.Dolei, AntoninaQin, ZhiqiangDomanovic, DragoslavQiu, Hua-JiDomańska-Blicharz, KatarzynaQu, BingqianDombrovsky, AvivQu, FengDomenech, AnaQuackenbush, SandraDomier, LeslieQuadeer, Ahmed A.Domingo, CristinaQuéré, PascaleDomingo, MarianoQuesada, ErnestoDomingo-Calap, PilarQuigley, Bonnie L.Donadu, MatthewQuiñones-Mateu, Miguel E.Donaire, LiviaRabalski, LukaszDonald, Claire L.Racine, TrinaDonato, CelesteRadke, Jay RDong, ShengzhangRadlinska, MonikaDoore, Sarah M.Radoshitzky, Sheli R.Dopazo, Carlos P.Ragan, IzabelaDorotkiewicz-Jach, AgataRaghavan, SudhirD'orso, IvanRagonnert-Cronin, ManonDosoky, NouraRagonnet-Cronin, ManonDoszpoly, AndorRagupathy, ViswanathDoty, JeffreyRahman, MotiurDou, XiaoguangRai, Devendra KumarDoublet, VincentRainey, StephanieDouglas, Mark W.Rajão, Daniela S.Dowd, Kimberly A.Rajarapu, Swapna PriyaDražić, MajaRajarathnam, KrishnaDrebot, MikeRajko-Nenow, PaulinaDrigo, MicheleRakonjac, JasnaDrillien, RobertRakotondrafara, AurelieDrolet, BarbaraRamanathan, PalaniappanDrozd, RadoslawRamette, AlbanDruce, JulianRamirez, HugoDrucker, MartinRanadheera, SenakaDrummer, HeidiRanawade, AyushDu, JuanRandazzo, WalterDu, KeRappocciolo, GiovannaDubé, MathieuRashed, ArashDubovi, EdwardRastelli, EugenioDucatez, MarietteRathinam, Navanietha KrishnarajDucatez, Mariette F.Rathore, Abhay PSDuchêne, SebastiánRatmann, OliverDudley, Jaquelin P.Rausch, Jason W.Dudman, Susanne G.Ravelonandro, MichelDugdale, BenjaminRay, RatnaDuke, ElizabethRayner, JonathanDunne, MatthewRazzuoli, ElisabettaDunowska, MagdalenaRead, AndrewDurand, Guillaume A.Rebollo, RitaDürrwald, RalfRedd, AndrewDutartre, HélèneReddy Bolla, JaniDutch, RebeccaReddy, SanjayDvorak, CherylReddy, VijayDyall, JulieReddy, Vishwanatha R.A.P.Dyall-Smith, MikeRedwood, AlecDykeman, EricRegla-Nava, Jose A.Dziewit, LukaszRegoes, RolandEastwell, Kenneth C.Reguera, JuanEbert, GregRegunath, HariharanEbrahimi, DiakoReichel, Michael P.Echevarría, Juan E.Reid, ScottEcke, ThorstenReid, St PatrickEckels, Kenneth H.Reid, StevenEden, John-SebastianReid, WilliamEdenborough, KathrynReina, RamsésEdgar, JamesReineke, LucasEdlefsen, Paul T.Reis, AnaEgberink, HermanRen, PingEggers, Christian HResa-Infante, PatriciaEggink, DirkRety, StephaneEgyed, LászlóReuscher, CarinaEhlers, BernhardRevill, PeterEhmann, RosinaReyes-Sandoval, ArturoEhrhardt, AnjaReynolds, MaryEhrlich, MarceloRichardson, JuliaEichwald, CatherineRichman, Douglas D.Elbadry, Maha ARichner, JustinEldar, AviRichner, Justin M.Elena, CriscuoloRicht, JuergenEl-Gazzar, Mohamed M.Riedel, ChristianeElghobashi-Meinhardt, NadiaRieder, ElizabethEl-Hage, NaziraRiera, MartaElliman, JenniferRigling, DanielEl-Mayet, FouadRiley, Kasandra J.Elofsson, MikaelRim, John HoonElsayed, Ahmed MostafaRimstad, EspenElshabrawy, Hatem A.Rinder, MonikaElshesheny, RabehRisch, MartinEmam, MehdiRissanen, IlonaEmerson, JoannaRittschof, ClareEndo, HisashiRivas, CarmenEngeland, Christine E.Riveiro-Barciela, MarEngland, MarionRivero-Juárez, AntonioEnjuanes, LuisRizvanov, AlbertEnosi Tuipulotu, DanielRobert, PhilippeErgunay, KorayRoberts, JohnEriko, OhsakiRobinson, ChrisErmonval, MyriamRobinson, KarlErtl, ReinhardRobinson, Sally R.Eschbaumer, MichaelRoby, Justin A.Escribano-Romero, EstelaRockett, Rebecca JEscriu, FernandoRockman, StevenEscudero-Abarca, BlancaRodo, CarlotaEscudero-Pérez, BeatrizRodriguez, FernandoEskelin, KatriRodríguez, FernandoEssani, KarimRodriguez-Andres, JulioEtebari, KayvanRodríguez-Calleja, Jose MaríaEtienne, LucieRodríguez-Díaz, JesúsEttayebi, KhalilRodriguez-Valera, FranciscoEvani, Shankar JaikishanRogier, BodewesEverett, Helen E.Rohrmann, George FEvermann, JimRomalde, Jesús L.Ewans, MatthewRomano, Patricia SilviaEwing, AdamRomerio, FabioEyre, NicholasRonca, ShannonFaaberg, KayRong, Chan KuanFabeni, LaviniaRong, LijunFablet, ChristelleRoot, Michael J.Faccini, SilviaRoques, PierreFaita, FrancescoRosa, Bruce A.Falendysz, Elisabeth A.Rosa, CristinaFalkenberg, ShollieRose, KarrieFalkenberg, Shollie M.Rosenthal, MariaFan, WenchunRoss, PerranFan, Yi-ChinRossetto, Cyprian C.Farenc, CarineRossi, GianpaoloFarkas, SilvieRossier, OmbelineFast, MarkRost, GergelyFaulk, Christopher D.Rostal, MelindaFaulkner, Geoffrey JohnRothenberger, SylviaFauver, JosephRothenburg, StefanFavia, GuidoRotondo, John CahrlesFavier, Anne-LaureRoubalova, KaterinaFehr, AnthonyRouillé, YvesFeiss, Michael G.Rousso, ItayFeliziani, FrancescoRouth, AndrewFeng, CongRouthu, Nanda KishoreFeng, NingguoRoux, SimonFera, DanielaRowland-Jones, SarahFerdinandy, BenceRowley, Paul AndrewFereres, AlbertoRoyo, Luis J.Ferguson, BrianRozek, WojciechFernández, LucíaRuaro, BarbaraFernandez, MelissaRückert, ClaudiaFernández-Alarcón, ClaudiaRudd, PennyFerrara, FrancescaRudra, ArnabFerreira, FernandoRueppell, OlavFerrer, IsidroRuggiero, GiuseppinaFerrero, DiegoRuggli, NicolasFerrés, MarcelaRuigrok, Rob W.H.Ferriol, InmaculadaRuiz-Garcia, Ana BelenFerron, FrançoisRuiz-Garcia, LeticiaFerry, TristanRumbou, ArtemisFeschotte, CédricRuml, TomasFibriansah, GunturRumlová, MichaelaFichet-Calvet, ElisabethRusanen, JuusoFiguerola, JordiRusconi, StefanoFilipič, BratkoRussell, RodFinetti-Sialer, Mariella M.Rut, WiolettaFinlaison, Deborah S.Ryabov, EugeneFiorentini, SimonaRybka, Jakub DaliborFischer, CarloRynda-Apple, AgnieszkaFischer, MelinaRyndock, EricFischer, NicoleRyu, SangryeolFiume, GiuseppeSaad, JamilFlannery, JohnSacco, Randy E.Flehmig, BertramSadaoka, TomohikoFleming, DamariusSaelens, XavierFlenniken, MichelleSafak, MahmutFlenniken, Michelle L.Saha, IpsitaFleury, HervéSahani, RajkumarFocosi, DanieleSahoo, Malaya KumarFolegatti, PedroSaitoh, AkihikoFoley, Brian T.Saitou, NaruyaFonseca, Gregory J.Sakai, HiroyasuFonseca, KevinSakamaki, IppeiFontana, JuanSakellariou, DikaiosFontanay, StéphaneSaksela, KalleFoppa, Ivo M.Saláková, MartinaFord, KathrynSalama, Sofie R.Forni, DiegoSalánki, KatalinForrest, CraigSalata, CristianoForrester, NaomiSaleh, Fatima A.Forsman, MatsSalman, M. D.Forstova, JitkaSalpini, RominaFortuna, ClaudiaSalter, Jason D.Forzan, MarioSalvato, MariaFossé, PhilippeSalvetti, AnnaFossen, TorgilsSamarasinghe, AmaliFoster, Toshana LauriaSamiotaki, MartinaFox, JulieSamy, AhmedFoxi, CiprianoSamy, RaviFrada, MiguelSanchez, David JesseFralick, Joe A.Sanchez, GloriaFrança, Monique S.Sanchez-Lockhart, MarianoFrancis, Ashwanth ChristopherSánchez-Madrid, FranciscoFranco, DavidSandro, RolesuFrancois, CatherineSang, YongmingFrançois, SarahSangster, Mark Y.Frangeul, LionelSankale, Jean-LouisFrank, LuizaSanna, GiuseppinaFrant, MaciejSano, TeruoFranzoni, GiuliaSansone, PasqualeFraune, SebastianSantiago-Rodriguez, Tasha MarieFreiberg, Alexander N.Santoro, Maria MercedesFreimanis, GrahamSantos, RicardoFrentiu, FrancescaSantoso, NettyFrese, MichaelSanz Muñoz, IvanFreuling, ConradSapp, MartinFrias Casas, MarioSarathy, Vanessa V.Froissart, RémySargueil, BrunoFrolova, ElenaSaribaş, Abdullah SamiFros, JelkeSarid, RonitFrossard, Jean-PierreSariol, CarlosFrunzke, JuliaSarker, SubirFrutos, RogerSarr, MoussaFu, Tong-MingSarute, NicolasFuchs, MarcSatheshkumar, Panayampalli SubbianFuji, Shin-IchiSato, HironoriFujikura, DaisukeSattentau, QuentinFujita, RyosukeSauder, ChristianFujiwara, KeiSaulle, IrmaFukuhara, TakasukeSauter, DanielFukushi, HidetoSautto, Giuseppe A.Fukuta, ShiroSavenkov, EugeneFukuzawa, Noriho H.Savic, VladimirFuller, Frederick J.Saxena, PoojaFuller, TrevonScallan, MartinaFurlini, GiulianoScarborough, Robert J.Furuse, YukiScaturro, PietroFuruya, TetsuyaSchachner, AdenaFuruya, YoichiSchairer, CynthiaFusco, DahleneScharf, BirgitGabriel, EmmanuelSchemmerer, MathiasGaffuri, AlessandraScheuermann, Richard H.Gaglia, Marta MariaSchildgen, OliverGagne, RoderickSchildgen, OlivierGajda, AnnaSchmidt, Aaron G.Galabov, AngelSchmidt, Peter ThelinGałas, AleksanderSchmidt, PhilipGale, PatrickSchmidtke, MichaelaGallagher, ThomasSchneider-Schaulies, JuergenGallardo, RodrigoSchneidman-Duhovny, DinaGambardella, ClaudioSchnierle, Barbara S.Gambarian, AlexandraSchoen, Robert T.Gambino, GiorgioSchols, DominiqueGammon, DonSchommers, PhilippGanaie, SafderSchönrich, GüntherGanges, LlilianneSchotta, GunnarGanji, RakeshSchreiber, MichaelGann, Eric R.Schrier, RachelGanor, YonatanSchroeder, DeclanGanser-Pornillos, BarbieSchrum, AdamGanser-Pornillos, Barbie K.Schubert, GritGao, YongSchubert, UlrichGarbuglia, Anna RosaSchuetz, AlexandraGarcía, PedroSchuler Faccini, LaviniaGarcia-Dorival, IsabelSchultz, DavidGarcia-Estañ, Luis PerezSchultz, ThomasGarcía-Murria, María J.Schulz, ClaudiaGarcia-Ruiz, HernanSchumpp, OlivierGardner, Matthew J.Schvoerer, E.Garfinkel, DavidSchwartz, David A.Garigliany, Mutien-MarieSchwarz, AnkeGarofalo, MariangelaSchweizer, MatthiasGarten, WolfgangSealy, JoshuaGascuel, OlivierSediva, AnnaGastaminza, PabloSeferovic, Maxim D.Gattinger, PiaSegalés, JoaquimGaudin, YvesSekiguchi, SatoshiGaudreault, Natasha NSekijima, Masakazu　Gauger, Phillip C.Seley-Radtke, KatherineGehrke, LeeSemino, CarlosGeisler, ChristophSemler, Bert L.Geldenhuys, MarikeSencanski, MilanGendrot, MathieuSeo, Sang HeuiGeneroso, Jaqueline Da SilvaSerra-Moreno, RuthGeng, TingtingSerwer, PhilipGerber, Priscilla F.Setny, PiotrGerhauser, IngoSewald, KatherinaGerlich, WolframSewald, XaverGershon, MichaelSgouralis, IoannisGhosh, SouvikShackelford, JuliaGhosh, SumitShah, PriyaGiadinis, Nektarios D.Shahi, Shailesh K.Giammanco, Giovanni MShamay, MeirGiangaspero, MassimoSharma, LokeshGiannecchini, SimoneSharp, ClaireGifford, RobertSharp, Natasha J.Gil, Jose FernandoSharshov, KirillGill, JasonShaw, AndrewGimenez-Lirola, Luis G.Sheehy, NoreenGinet, NicolasSherer, NathanGiovanardi, DavideShestopalov, Alexander MGish, RobertShi, MangGiuffrida, PaoloShiba, KiyotakaGladue, DouglasShih, ChiahoGlasa, MiroslavShim, HyunboGlenn, WendyShimoda, MichikoGlowacz, AdamShimojima, MasayukiGoffinet, ChristineShimura, HanakoGoh, Gerard Kian-MengShin, Hyun-JinGoldbourt, AmirShin, Ok SarahGoldstein, RichardShinkai, YoichiGoldstein, RonaldShioda, TatsuoGolec, PiotrShiokawa, MaiGolender, NataliaShirai, JyunsukeGolender, NatalyaShirato, HarukoGoletti, DeliaShisler, JoannaGolubchik, TanyaShivanna, VinayGolubovskaya, VitaShpacovitch, VictoriaGómez Castilla, JordiShriner, SusanGomez Monterrey, IsabelShukla, DeepakGomez-Lucia, EsperanzaSicheritz-Pontén, ThomasGong, BinSiddappa, ByrareddyGong, PengSiddharthan, VenkatramanGonzales, Jose LSieben, ChristianGonzalez, GabrielSignorini, LuciaGonzalez, Jean-PaulSilva, RicardoGonzalez, John-PaulSilva, WalterGonzález-Bulnes, AntonioSilveira, CynthiaGonzalez-Dunia, DanielSimon, FrançoisGonzalez-Reiche, Ana SilviaSimpson, David J.Goodier, Martin R.Sin, JonGoodman, Alan G.Sinderewicz, EmiliaGoodman, Cynthia L.Singanalur, Nagendrakumar BalasubramanianGoodwin, ThomasSingh, GurdeepGossner, Céline Marie EliseSinigaglia, AlessandroGouandjika-Vasilache, IonelaSinkins, StevenGozalbo Rovira, Roberto VicenteSioofy-Khojine, AmirbabakGralinski, LisaSironen, TarjaGrand, RogerSistla, SrinivasGrandi, NicoleSkelly, DeanneGrant, MichaelSkurnik, MikaelGreen, KimSlavcev, RoderickGreen, ToddŚliwińska-Wilczewska, SylwiaGreiner, TimoSloan, ChantelGreive, SandraSluis-Cremer, NicolasGriffin, HeatherSmartt, Chelsea T.Griffiths, David J.Smerdou, CristianGrimsley, NigelSmith, BruceGrose, JulianneSmith, DonaldGrose, Julianne H.Smith, DougGrove, JoeSmith, GregoryGroves, IanSmith, JessicaGrubman, MarvinSmith, Lauren M.Grünweller, ArnoldSmith, ThomasGu, ZhenSmith, Todd G.Guardado-Calvo, PabloSmithgall, Thomas E.Guerra-Assuncao, AfonsoSmodiš Škerl, MajaGuerrero-Plata, AntonietaSmura, TeemuGuerrini, MarcoSnoeck, Chantal J.Guido, PoliSnow, Jonathan W.Guillaume, MousseauSöderberg-Naucler, CeciliaGuillon, ChristopheSöderlund-Venermo, MariaGuo, Ming-LeiSohn, SeonghyangGuo, Zong ShengSokoloski, KevinGupta, KapilSokolova, Olga S.Gustin, KurtSolana, RafaelGutiérrez-López, RafaelSolis, MorganeGuy, BrunoSollai, GiorgiaGuy, Paul L.Solmaz, SozanneGuydosh, Nicholas R.Solovyev, AndreyGyőző, KajánSompuram, SeshiGysi, DeisySong, JianxunGyula, PéterSong, Min-SukHaan, Peter DeSong, Moon JungHaase, Astrid D.Sooryanarain, HariniHabili, NuredinSoriano, VicenteHadidid, AhmedSoudeyns, HugoHadziyannis, EmiliaSousa, AngelaHaese, Nicole N.Souza, MeniraHagiwara, KatsuroSozzi, EnricaHahm, BumsukSpada, EvaHahn, AlexanderSparger, ElizabethHai, RongSpathis, Aris T.Hairgrove, ThomasSpatz, StephenHajarizadeh, BehzadSpeck, Peter G.Hakami, RaminSpengler, Jessica R.Hakata, YoshiyukiSpiegel, MartinHaley, Sheila A.Spiro, David J.Hall, Roy A.Sridhar, SiddharthHallden, GunnelStadejek, TomaszHall-Mendelin, SonjaStafford, Graham P.Hamel, RodolpheStainton, DaisyHammarskjöld, Marie-LouiseStamminger, ThomasHammond, JohnStanifer, Megan L.Hampson, LynneStanton, James B.Han, Hui-JuStapleford, KennethHan, LeiStapleford, Kenneth A.Hang, JunStapleton, JackHankaniemi, Minna M.Stavolone, LiviaHanks, Ephraim M.Stavrou, SpyridonHann, Hie-WonSteel, Laura F.Hans, AymericSteger, GerhardHaqshenas, GholamrezaSteger, GertrudHaque, MuzammelSteinbach, FalkoHarder, TimmSteinmann, EikeHardies, Stephen C.Stepanova, EkaterinaHarding, RobStephens, EdwardHarmon, Karen M.Sternberg-Lewerin, SusannaHarper, David R.Stevanovic, JevrosimaHarrach, BalázsSteyer, Dr. AndrejHarrich, DavidStobart, ChristopherHarris, AudrayStone, DanielHarris, ReubenStoye, Jonathan PaulHarrison, RobertStrand, Micheline K.Harrod, RobertStrecker, ThomasHartert, Tina V.Strong, JimHartshorn, Kevan LStrutt, Tara M.Hartung, Hans-PeterStuart, PatrickHarty, RonaldStubenrauch, FrankHarvey, WilliamStukelj, MarinaHassan, Sharifah SyedSu, WeihengHatano, YuichiroSuárez, Nicolás M.Hatfull, GrahamSubbiah, JeevaHattori, ToshioSubr, ZdenoHause, BenŠubr, ZdenoHawes, PhilippaSucena, ÉlioHay, Iain DSuenaga, HikaruHayasaka, DaisukeSuffredini, ElisabettaHayer, JulietteSukumaran, SunithaHayes, Sidney J.Sullivan, Brian M.Hazan, RonenSullivan, ChrisHe, JieSulzenbacher, GerlindHe, Mike Z.Summers, MichaelHe, QigaiSun, CaijunHearing, PatrickSun, HaiyanHébrard, EugénieSun, XiangjieHedman, KlausSun, XiaomingHefferon, Kathleen L.Surapathrudu, KanakalaHefferon, Kathleen LauraSusi, PetriHegele, Richard GSutton, RichardHeinlein, ManfredSutummaporn, KripitchHeitz, ThierrySuzuki, TohruHejnar, JiriSvircev, AntonetHelbig, KarlaSweeney, TrevorHelle, FrancoisSwetnam, Daniele M.Henderson, AndrewŚwiętoń, EdytaHenderson, Andrew J.Switzer, WilliamHeng, XiaoSzakal, EvelinHenke, AndreasSzczepankowska, Agnieszka K.Henrich, Curtis J.Sztanke, KrzysztofHepojoki, JussiSzymczak, AleksandraHerchenröder, OttmarTada, RuiHerker, EvaTagad, Harichandra D.Hernáez, BrunoTai, WanboHernández, CarmenTai, William Chi-ShingHernandez, JesusTajima, MotoshiHernandez-Vargas, EstebanTakada, AyatoHernandez-Vargas, Esteban A.Takahashi, TomokoHerod, MorganTakano, TomomiHerrera-Carrillo, ElenaTakeda, ShigekiHersperger, Adam R.Takemura, MasaharuHertel, RobertTakeuchi, RikiyaHerzog, CatherineTakeuchi, YasuhiroHeselpoth, RyanTakimoto, ToruHesson, JennyTakizawa, NaokiHicks, Julie A.Talker, StephanieHijano, Diego R.Tamba, MarcoHildt, EberhardTamori, AkihiroHill, FraserTan, WenbinHillyer, JulianTanaka, YoshikazuHily, Jean-MichelTandon, RiteshHingamp, Pascal M.Tang, QiyiHinkula, JormaTaniguchi, KokiHinton, DeborahTaniguchi, SatoshiHipp, KatharinaTao, PanHirayama, KazuhiroTarasova, Ol'Ga A.Hirono, IkuoTarlinton, RachaelHirota, JeremyTaskinen, SaraHirsch, Alec J.Tate, MichelleHirsch, HansTate, Shin-IchiHirsch, IvanTatsuya, KatoHirsch, JudithTattersall, PeterHirsch, Silvia AyoraTaube, StefanHizi, AmnonTauriainen, SiskoHo, Chak-SumTaus, NaomiHo, Eric S.Tavanti, FrancescoHo, Ya-ChiTaylor, AdamHobson-Peters, JodyTaylor, IanHodcroft, Emma B.Te Velthuis, AartjanHoeben, RobTedbury, PhilipHoek, Lia Van DerTelenitsky, AliceHoenen, ThomasTempera, ItaloHoffmann, MarkusTempesta, MariaHöfle, UrsulaTenenbaum, TobiasHofstetter, Amelia R.Teng, Lee LeeHogan, Michael J.Teramoto, TadahisaHogue, IanTeras, RihoHolbrook, MichaelTerio, ValentinaHolicki, Cora M.Terranova, CorradoHollister, Emily B.Terryn, SanneHolmes, Edward C.Teschke, CarolynHölzer, MartinTessarz, PeterHolzhauer, MennoTestoni, BarbaraHoma, Fred L.Thali, MarkusHong, XupengThanki, Anisha MahendraHopken, Matthew W.Theuns, SebastiaanHorhat, Florin GeorgeThiebes, Anja LenaHorie, MasayukiThiry, EtienneHorimoto, TaisukeThomas, John C.Horton, NancyThomas, JulieHorton, Nancy C.Thompson, Andrew J.Horwood, Paul FThompson, JeremyHosmalin, AnneThwaites, Ryan S.Hostnik, PeterTian, YanpingHou, YixuanTietjen, IanHouldcroft, CharlotteTikoo, Suresh K.Houston, FionaTimofeev, Vladimir I.Hovi, TapaniTischer, KarstenHraber, PeterToborek, MichalHribar, LawrenceTodt, DanielHristov, Peter IvanovToffan, AnnaHsieh, Ming KunTognon, MauroHsieh, Shie-LiangTohma, KentaroHu, JiafenTollenaere, CharlotteHu, YanmeiTolstrup, MartinHuang, ChengTonelli, MicheleHuang, ChienjinTonetti, Michela G.Huang, Hsing ITong, ShupingHuang, Jason C.Toniolo, Antonio Q.Huang, YongTonni, GabrieleHuang, ZiweiToplak, IvanHubai, AndrásTorcia, Maria GabriellaHuber, AndrewTorre, MarialuisaHuber, VictorTorres, AliceHufbauer, MartinTortorella, DomenicoHufton, Simon E.Torzewska, AgnieszkaHughes, Holly R.Toth, KarolyHughes, JosephToubanaki, DimitraHughes, StephenToussaint, ArianeHume, AdamTouzé, AntoineHummel, MaryTrabaud, Mary-AnneHurst, ChristonTrapp, SaschaHutson, ChristinaTrapp-Fragnet, LaëtitiaHwang, Eung-SooTravis, JohnHynes, AlexanderTraylor-Knowles, NikkiHyodo, KiwamuTreder, KrzysztofHytönen, VesaTremblay, NicolasIbrahim, AhmedTripp, Ralph A.Ikebuchi, RyoyoTrobaugh, DerekIkeda, TerumasaTroyer, RyanImai, MasakiTrubl, GaryImbeault, MichaelTruncaitė, LidijaIncarnato, DannyTruniger, VerónicaIndugu, NagarajuTrus, IvanInoue, JunTsai, Chi-WeiIordanskiy, SergeyTsai, Jih-JinIorio, Ronald M.Tsai, Ming-HanIsakova-Sivak, IrinaTsetsarkin, Konstantin A.Isel, CatherineTso, For YueIseni, FrédéricTsourkas, PhilipposIshibashi, KazuhiroTsuge, HideakiIshikawa, MasayukiTsukamoto, TetsuoIshikawa, TomohiroTsukiyama-Kohara, KyokoIshima, RiekoTsumoto, KantaIshizaki, AzumiTu, ThomasIskra-Caruana, Marie-LineTucci, JosephIslam, ArifulTullman-Ercek, DanielleIto, KimihitoTully, DamienIto, NaotoTuma, RomanIturriza-Gomara, MirenTumban, EbenezerIvankovic, TomislavTun, Mya Myat NgweIwano, HidetomoTungadi, TrisnaIwanowicz, Luke RTuomivirta, TeroIwasaki, MasaharuTurchi, ChiaraIzsvák, ZsuzsannaTurina, MassimoJ. Jackwood, DaralTurnbull, Matthew W.Jääskeläinen, Anne J.Turner, DannJackova, AnnaTwarock, ReidunJackson, BrianTworowski, DmitryJackson, Kathy M.Tyagi, MuditJackson, Sarah E.Tyrrell, D LorneJacob, GopasUcciferri, ClaudioJacobs-Sera, DeborahUeda, KeijiJadhao, Samadhan J.Uehara-Ichiki, TamakiJaguva Vasudevan, Ananda AyyappanUgaki, MasashiJaimes, Javier A.Ulaeto, DavidJakab, FerencUlbert, SebastianJakimovski, DejanUlrich, RainerJakobsson, JohanUlrich, ReinerJalava, KatriUmasuthan, NavaneethaiyerJames, BurkeUnzai, SatoruJames, Claire DUpadhyay, ChitraJames, WilliamUpadhyay, SandeepJamin, MarcUpton, ChrisJan, EricUrayama, ShunichiJan, Fuh-JyhUrbanelli, LorenaJancovich, JamesUrra, José MiguelJanelle, ValerieUsmani, ShariqJangra, RohitUsui, TatsufumiJanowski, Andrew B.Uversky, VladimirJansen, C.A. (Christine)V Ivanov, AlexanderJansen, StephanieVaira, Anna MariaJardine, PaulVales-Gomez, MarJarocki, PiotrValiakos, GeorgeJarosinski, KeithValkenburg, Sophie A.Jeger, MichaelValles, StevenJenckel, MariaVan Baalen, MinusJenkins, FrankVan Borm, StevenJeonga, Dae GwinVan Bortel, WimJeske, HolgerVan De Klundert, MaartenJiang, GuochunVan De Vijver, David AMCJiang, Qiu-XingVan Den Hoogen, BernadetteJiang, ShijinVan Den Hurk, AndrewJiang, SizunVan Den Wollenberg, Diana J. M.Jiménez De Oya, NereidaVan Der Hoek, LiaJin, Dong-YanVan Der Kuyl, AntoinetteJin, JingVan Der Poel, WimJin, TengchuanVan Der Sluis, Renée M.Jin, YuefeiVan Doorslaer, KoenraadJochmans, DirkVan Engelenburg, SchuylerJõers, PriitVan Etten, JamesJohn, Sinu P.Van Gils, Marit J.Johnson, JohnVan Lint, CarineJohnson, NicholasVan Marle, GuidoJohnson, Reed F.Van Raaij, MarkJohnston, J. SpencerVan Rijn, PietJohnston, Randal N.Van Santen, VickyJończyk-Matysiak, EwaVandeWoude, SueJones, Bryony A.Vaney, Marie-ChristineJones, ClintonVanham, GuidoJones, IanVanlandingham, DanaJones, JeremyVanpouille, ChristopheJones, MelissaVarallyay, EvaJordan, BrianVarjak, MargusJordan, Jeanne A.Varsani, ArvindJordan, KingVassaux, GeorgesJordan, Michael RVasse, MarcJørgensen, Charlotte SværkeVassilaki, NikiJose, JoyceVassilakos, NikonJoshi, HirenVázquez-Domínguez, EvaristoJosset, LaurenceVecchio, EleonoraJulander, Justin G.Velasco, LeonardoJung, AlainVelasco-Villa, AndrésJung, Yong SamVenuprasad, PoojaryJurak, IgorVerchot, JeanmarieJurvansuu, JaanaVernick, Kenneth D.Kaczmarek, Maria E.Vérollet, ChristelKaczorowski, TadeuszVerrier, Eloi RKading, Rebekah C.Viana, MafaldaKaelber, JasonVidana Mateo, BeatrizKafri, TalViejo-Borbolla, AbelKageyama, TsutomuVijayan, MadhuvanthiKainulainen, Markus HVilcek, StefanKaján, Győző L.Vilibić-Čavlek, TatjanaKajihara, MasahiroVimalanathan, SelvaraniKalinowski, JörnVinje, JanKallies, ReneViranaicken, WildrissKamau, EverlynVisalli, Robert J.Kanda, TatsuoVisseaux, BenoitKanda, TeruVitour, DamienKandeel, MahmoudVivet-Boudou, ValérieKang, Sang-MooVlasova, Anastasia N.Kant, SashiVogels, ChantalKantor, AsherVogt, Peter K.Kaplan, ShaunaVolchkova, ValentinaKarimi, KhalilVolle, RomainKarlsson, ErikVolz, AsisaKarniychuk, UladzimirVon Dobschuetz, SophieKároly, FátyolVon Laer, DorotheeKarsten, Christina BeatriceVranken, WimKaruppannan, Anbu KumarVu, LuanKashanchi, FatahVujadinovic, MarijaKashiwagi, AkikoVuorinen, TyttiKathuria, HimanshuWadell, GöranKatneni, UpendraWagemans, JeroenKatoh, HiroshiWakimoto, HiroakiKatsoulos, Panagiotis D.Waldorf, Kristana AdamsKatz, RichardWalker, Kathleen RKatzenellenbogen, Rachel A.Walker, Peter J.Kaufer, Benedikt B.Walther, PaulKaufmann, AndreasWan, HongquanKaul, MarcusWan, WilliamKautz, Tiffany F.Wang, BoKawada, Jun-ichiWang, ChihyangKay, BrianWang, Ching-HoKayali, GhaziWang, Chin-TienKazama, ShinobuWang, DavidKe, Po-YuanWang, Fun-InKearney, Mary F.Wang, Hao-ChingKedzierska, KatherineWang, HURNG-YIKehn-Hall, KyleneWang, JinKeita, AlphaWang, JiongweiKelch, BrianWang, JuexinKell, AlisonWang, JunKelly, GabrielleWang, LeyiKemenesi, GáborWang, LingKennedy, MelissaWang, NianshuangKennedy, Richard B.Wang, RichardKenney, Joan L.Wang, Robert Y.-L.Kenney, Scott P.Wang, XiaohongKhaperskyy, Denys A.Wang, XiuqinKhatri, VishalWang, YingKhayat, RezaWang, ZhengqiangKheimar, AhmedWang, ZiwenKhurshid, ZohaibWang, ZongjieKibenge, FrederickWarner, NadiaKibler, Karen V.Warrell, MaryKiefer, DorotheeWarrilow, DavidKiełpińska, JolantaWasniewski, MarineKieran, Troy JWatanabe, SatoruKierzek, ElzbietaWatanabe, ShumpeiKiga, KotaroWatanabe, TokikoKil, Eui-JoonWebster, CraigKile, James C.Weger, StefanKillian, Mary LeaWegrzyn, AlicjaKilpatrick, MarmWegrzyn, GrzegorzKim, ChonsaengWęgrzyn, GrzegorzKim, Dal YoungWei, DongqingKim, Hye KwonWei, HongpingKim, In-JeongWei, YonglongKim, Jee HyunWeidner-Glunde, MagdalenaKim, JoomyeongWeinbauer, MarkusKim, Ki-HongWeiser, RebeccaKim, MeehyeinWeissenhorn, WinfriedKim, MikyeongWeitzman, MatthewKim, SeilWelch, Stephen RKim, Su-miWelsh, MichaelKimpel, JanineWen, Chiu-MingKimura, HirokazuWenping, QiuKincaid, RodneyWertheim, JoelKinchington, PaulWestman, MarkKing, DonaldWeyenbergh, Johan VanKing, LindaWeynberg, KarenKingsley, DavidWhitby, DeniseKipar, AnjaWhite, JohnKirchhoff, FrankWhite, JudithKireev, DmitryWhite, KirstenKirkwood, CarlWhite, Peter A.Kirubakaran, PalaniWhite, SimonKirui, JamesWhitford, Paul CKis, ZoltanWhitney, James B.Kiseleva, Irina V.Whitney, JohnKishida, YutakaWichgers Schreur, Paul J.Kiso, MakiWigdahl, BrianKitchen, AndrewWilke, AndreKlapper, PaulWilkes, Rebecca P.Klase, Zachary A.Willett, BrianKlein, Terry A.Wilson, AngusKleinow, TatjanaWilson, Carolyn A.Kleinpeter, AlexWilson, Van G.Klenk, Hans-DieterWilson, WilliamKlimkait, ThomasWilson, William C.Klonjkowski, BernardWilusz, JeffKlose, ThomasWinkler, Michael E.Klyczek, KarenWinter, Deborah RachelleKnepper, JaniceWira, CharlesKnight, Richard J.Wise, Annabel G.Knowles, GraemeWise, Emma L.Knowles, Nick J.Witkowski, WojciechKo, Eun-juWold, William S.M.Kobayashi, TetsuroWolf, StevenKobie, James J.Wölfel, RomanKobiler, OrenWollebo, Hassen S.Kochneva, GalinaWong, GraceKock, RichardWong, Richard W.Kodidela, SunithaWong, Sek-ManKoethe, SusanneWood, BrittaKohara, MichinoriWorkman, Aspen M.Koivisto, JanneWoźniakowski, GrzegorzKolawole, AbimbolaWright, DanielKolbasov, DenisWu, Fang-TzyKolishetti, NageshWu, GuanghuiKomar, NicholasWu, I-ChinKomatsu, KenWu, JianyongKomorowska, BeataWu, Suh-ChinKondili, LoretaWu, WenxinKondo, HidekiWu, XuelingKondo, SatoruWu, YanlingKönig, AlexanderWu, YuntaoKonjevic, DeanWylie, KristineKoodie, LisaXavier, CatarinaKoos, BjörnXia, NingshaoKordyukova, LarisaXia, YuchenKormelink, RichardXian, YuejiaoKormuth, KarenXiao, YongliKoromyslova, AnnaXie, DeyuKorth, JohannesXie, JiataoKoshiba, TakumiXie, JiexiongKoshizuka, TetsuoXin, YinKosik, IvanXu, JianKoskan, AlexisXu, PeiKoslová, AnnaXu, WenxingKostaki, EvangeliaXue, GuangaiKostyushev, DmitryYamamoto, MasatoKosulin, KarinYamamoto, TakehisaKotta-Loizou, IolyYamanaka, AtsushiKouyos, Roger DYamaoka, SatokoKovarik, AlesYamauchi, YoheiKozak, ChristineYamayoshi, SeiyaKozieł, EdmundYanase, TohruKoziol-White, Cynthia J.Yang, Hung-ChihKozlovskaya, LiubovYang, KuiKozlowski, LukaszYang, SiyoungKrammer, FlorianYang, XiulingKraska, ThorstenYao, YongxiuKrejbich-Trotot, PascaleYasuike, MotoshigeKrenz, BjörnYau, ShereeKrishnakumar, DevadasYe, XiaohuaKrishnamurthy, Siddharth R.Yeager, MeredithKroeker, AndreaYeh, Hsin-HungKrol, EwelinaYehl, KevinKrug, LaurieYellapu, Nanda KumarKrug, PeterYen, Pei-ShiKruger, DetlevYin, JohnKrüger, NadineYin, LiKruse, Robert L.Yin, RenfuKryger, PerYin, ZhiminKrylov, VictorYlösmäki, ErkkoKrzyzowska, MalgorzataYolande, RichardKubo, YoshinaoYoldi, Miguel Julián MartínezKucheryavykh, Lilia Y.Yoneyama, MitsutoshiKugelman, Jeffrey R.Yoo, Ji YoungKuhar, UrškaYoo, So YoungKula-Pacurar, AnnaYoon, HachungKulkarni, AmolYoon, Sun-WooKumar Kundu, JibanYoshida, EricKumar, AmitYoshimatsu, KumikoKumar, AshokYost, Christian ConKumar, AsitYouk, SungsuKumar, BinodYoung, Howard A.Kumar, MukeshYoung, RylandKumar, PenmetchaYount, Jacob S.Kumar, ShaileshYousef, AhmedKumari, AshaYu, JingyouKümmerer, Beate M.Yu, Xiao-FangKunec, DusanYu, Xue-JieKuniya, ToshikazuYuan, HaoKuo, Yen-WenYuan, ShuofengKurebayashi, YuukiYuchun, DuKurian, Justin J.Yue, Xiao-GuangKurt, ToblerYuen, LillyKüry, PatrickYuh, Chiou-HwaKuryk, LukaszYuill, ThomasKusejko, KatharinaYura, YoshiakiKuss-Duerkop, Sharon K.Zachou, KalliopiKuzmak, JacekZago, MiriamKuzmin, IvanZaid, AliKuzmina, Natalia A.Zakhartchouk, AlexanderKvale, DagZamperin, GianpieroKwiatek, OlivierZanella, IsabellaKwilas, StevenZanier, KatiaKwon, AmyZanusso, GianluigiKwon, SunohZapata, JuanKwun, Hyun JinZecchin, BiancaKyei, George BZeev-Ben-Mordehai, TzviyaL. Schmaljohn, AlanZehender, GianguglielmoLa Rosa, GiuseppinaZeichner, Steven L.Laanto, ElinaZell, RolandLabo, NazzarenaZeltins, AndrisLacey, Charles J. N.Zeman, PetrLagatolla, CristinaZeng, LanyingLagaudrière-Gesbert, CécileZhang, BoLagunoff, MichaelZhang, DayiLai, Michael M. C.Zhang, GongyiLaksono, BrigittaZhang, Hong-YuLamb, LauraZhang, HuanminLamba, DorianoZhang, QiongLaMere, SarahZhang, RuiLanave, GianvitoZhang, ShuoLang, AndrewZhang, WenliLang, FengchaoZhang, YinfengLange, UlrikeZhang, YongLangel, Stephanie NealZhang, ZhenfengLangsjoen, RoseZhang, ZhidongLanteri, GiovanniZhang, ZhifangLattorff, H. Michael G.Zhao, JianjunLauzi, StefaniaZhao, JunLaval, KathlynZhao, QinjianLawrence, Charles M.Zhao, Xue ZhiLazar, CatalinZheng, Du-PingLe Goffic, RonanZheng, WenshuLe Mercier, PhilippeZheng, XuexingLe Pendu, JacquesZheng, ZhenhuaLe Sage, ValerieZheng, Zhi-MingLeal, Ana MendesZheng, Zi-ZhengLean, Fabian Z. X.Zhirnov, OlegLebrand, AitanaZhong, ZhaohuaLednicky, JohnZhou, AiminLee, Joo-YounZhou, DongmingLee, KellyZhou, Grace GLee, Keun HwaZhou, Heshan SamLee, Kuo HaoZhou, PengLee, Min YoungZhou, ShuntaiLee, Tzong-ShyuanZhu, JamesLeeks, AsherZhu, JianLeeuw, Erik DeZhu, QiyunLefkowitz, Elliot J.Zhu, XueyongLegendre, MathieuZidovec Lepej, SnjezanaLegler, PatriciaZienius, DainiusLehman, Dara A.Zimmer, GertLehman, John M.Zimmerman, JeffreyLeifels, MatsZmora, PawelLeis, Jonathan P.Żok, TomaszLeisi, RemoZoll, JanLeitão, AlexandreZolnik, Christine P.Lemay, GuyZou, WeiLemmermann, Niels A WZumbrun, ElizabethLenz, OndřejŽunec, RenataLeon-Juarez, MoisesZúñiga, ManuelLequime, SebastianZuñiga, SoniaLeroux, CarolineZuriaga, ElenaLesbats, PaulZüst, RolandLester, Philip JohnZwick, Michael B.Letarov, Andrey


